# Host Fatty Acid Utilization by Staphylococcus aureus at the Infection Site

**DOI:** 10.1128/mBio.00920-20

**Published:** 2020-05-19

**Authors:** Matthew W. Frank, Jiangwei Yao, Justin L. Batte, Jessica M. Gullett, Chitra Subramanian, Jason W. Rosch, Charles O. Rock

**Affiliations:** a Department of Infectious Diseases, St. Jude Children’s Research Hospital, Memphis, Tennessee, USA; University of Pittsburgh School of Medicine

**Keywords:** *Staphylococcus aureus*, fatty acid, fatty acid kinase, fatty acid binding protein, phospholipid, virulence, fatty acid activation, host-pathogen interactions

## Abstract

Staphylococcus aureus utilizes the fatty acid (FA) kinase system to activate exogenous FAs for membrane synthesis. We developed a lipidomics workflow to determine the membrane phosphatidylglycerol (PG) molecular species synthesized by S. aureus at the thigh infection site. Wild-type S. aureus utilizes both host palmitate and oleate to acylate the 1 position of PG, and the 2 position is occupied by pentadecanoic acid arising from *de novo* biosynthesis. Inactivation of FakB2 eliminates the ability to assimilate oleate and inactivation of FakB1 reduces the content of saturated FAs and enhances oleate utilization. Elimination of FA activation in either Δ*fakA* or Δ*fakB1* Δ*fakB2* mutants does not impact growth. All S. aureus strains recovered from the thigh have significantly reduced branched-chain FAs and increased even-chain FAs compared to that with growth in rich laboratory medium. The molecular species pattern observed in the thigh was reproduced in the laboratory by growth in isoleucine-deficient medium containing exogenous FAs. S. aureus utilizes specific host FAs for membrane biosynthesis but also requires *de novo* FA biosynthesis initiated by isoleucine (or leucine) to produce pentadecanoic acid.

## Abstract (precis)

The shortage of antibiotics against drug-resistant Staphylococcus aureus has led to the development of new drugs targeting the elongation cycle of fatty acid (FA) synthesis that are progressing toward the clinic. An objection to the use of FA synthesis inhibitors is that S. aureus can utilize exogenous FAs to construct its membrane, suggesting that the bacterium would bypass these therapeutics by utilizing host FAs instead. We developed a mass spectrometry workflow to determine the composition of the S. aureus membrane at the infection site to directly address how S. aureus uses host FAs. S. aureus strains that cannot acquire host FAs are as effective in establishing an infection as the wild type, but strains that require the utilization of host FAs for growth were attenuated in the mouse thigh infection model. We find that S. aureus does utilize host FAs to construct its membrane, but host FAs do not replace the requirement for pentadecanoic acid, a branched-chain FA derived from isoleucine (or leucine) that predominantly occupies the 2 position of S. aureus phospholipids. The membrane phospholipid structure of S. aureus mutants that cannot utilize host FAs indicates the isoleucine is a scarce resource at the infection site. This reliance on the *de novo* synthesis of predominantly pentadecanoic acid that cannot be obtained from the host is one reason why drugs that target fatty acid synthesis are effective in treating S. aureus infections.

## INTRODUCTION

**FIG 1:**
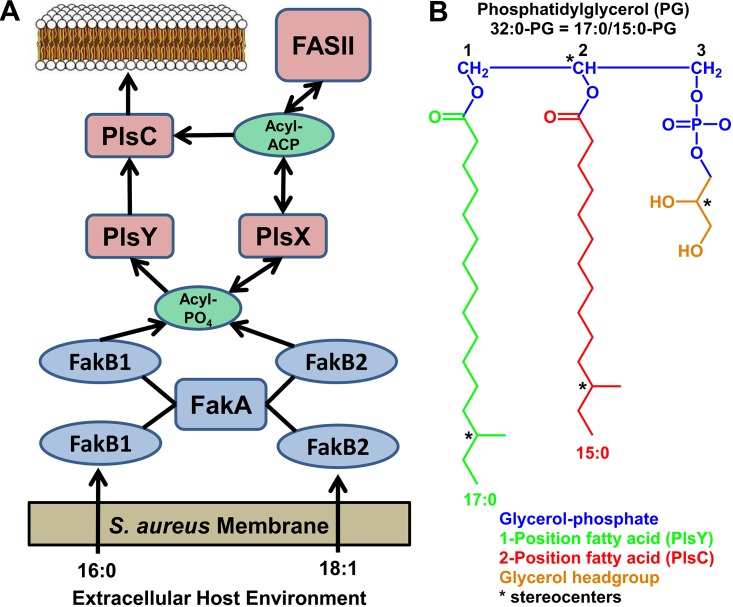
Model for the utilization of host FAs for phosphatidylglycerol (PG) synthesis by S. aureus. (A) FA kinase is the only pathway for the activation of extracellular FAs in S. aureus. Host FAs bind to one of the two FA binding proteins. FakB1 specifically binds saturated FA (16:0), and FakB2 selectively binds monounsaturated FA (18:1). The FakB(FA) complexes are phosphorylated by FakA, converting host FAs to the key intermediate, acyl-PO_4_. The resulting acyl-PO_4_ has two fates: PlsX and PlsY. PlsX converts acyl-PO_4_ to acyl-ACP that is elongated by FASII before being converted again to acyl-PO_4_ by PlsX. FASII produces 15:0 ACP that is preferentially used by PlsC to acylate the 2 position. (B) Structure of PG. The major membrane phospholipid of S. aureus consists of a glycerol phosphate backbone (blue) that is first acylated in the 1 position by acyl-PO_4_-dependent PlsY (green) followed by acylation of the 2 position by 15:0-ACP-selective PlsC (red). The glycerol headgroup (gold) is added by a series of three enzymes. There are four stereocenters (*) in the example show the abundant 17:0/15:0 PG molecular species (32:0 PG; *m/z *721) of S. aureus.

Three enzymatic systems to activate extracellular fatty acids (FAs) to initiate their downstream metabolism exist in bacteria (1, 2). FAs are converted to acyl-acyl carrier protein (ACP) by acyl-ACP synthetase, to acyl coenzyme A (acyl-CoA) by acyl-CoA synthetase and to acyl-phosphate (acyl-PO_4_) by FA kinase. Escherichia coli possess an acyl-CoA synthetase that activates FAs for incorporation into phospholipids or for degradation by β-oxidation (3). E. coli is a special case because its acyltransferases use either acyl-CoA or acyl-ACP as acyl donors, but most bacterial acyltransferases are either acyl-ACP or acyl-PO_4_ specific (4). Many Gram-negative bacteria (*Neisseria*, *Chlamydia*, and *Vibrio*) use acyl-ACP synthetases to activate FAs (5–7). In these cases, the acyl-ACP may be used by the acyltransferases or may be elongated by bacterial type II FA biosynthesis (FASII). Staphylococcus aureus and other Gram-positive bacteria use the FA kinase system as the only pathway for exogenous FA incorporation (Fig. 1A). This system consists of a kinase domain protein (FakA) that phosphorylates a FA bound to a FA binding protein (FakB) to form acyl-PO_4_ (8). S. aureus has two FA binding proteins. FakB1 specifically binds palmitate (16:0) and FakB2 is designed to bind oleate (18:1) (8–10) (Fig. 1A). The acyl-PO_4_-dependent PlsY acylates the 1 position of glycerol phosphate in the first step in phospholipid synthesis followed by acylation of the 2 position by the acyl-ACP-dependent PlsC (Fig. 1B). The product of the PlsY/C pathway (phosphatidic acid) is converted in three steps to the major S. aureus membrane phospholipid, phosphatidylglycerol (PG) (Fig. 1B). Acyl-PO_4_ derived from host FAs may either be incorporated into the 1 position by PlsY or be transferred to acyl-ACP by PlsX (phosphate:acyl-ACP transacylase), elongated by FASII, converted to acyl-PO_4_ by PlsX and then incorporated into the 1 position by PlsY (Fig. 1A). FASII of S. aureus produces pentadecanoic acid (15:0) that is selectively placed into the 2 position by PlsC (11, 12). The precursor to 15:0 is isoleucine (or leucine). S. aureus has a *de novo* biosynthetic route to Ile and Leu and efficiently uses extracellular branched-chain amino acids (13). Extracellular Ile or Leu is transaminated by IlvE (14) and converted to 2-methylbutyryl- or isovaleryl-CoA (C_5_-CoA) by branched-chain ketoacid dehydrogenase (15, 16). The C_5_-CoA is used by the FabH condensing enzyme to initiate FASII. This pathway results in a membrane phospholipid molecular species distribution with 15:0 in the 2 position of virtually all molecules and a variety of longer even- or odd-numbered FAs in the 1 position (11).

The importance of membrane lipid synthesis to bacterial physiology has led to the development of antimicrobial drugs targeting different steps in the FASII pathway (17–21). A major drug candidate against S. aureus is AFN-1252 (Debio1452/afabicin), which targets the enoyl-ACP reductase (FabI) component of FASII (11, 22–26). AFN-1252 effectively treats S. aureus infections in animal models (23, 24) and has completed two successful human phase II clinical trials (27, 28). A major objection to the use of FASII inhibitors to treat infections is that bacteria would bypass FASII inhibition by utilizing host FAs (29, 30). Although this may be an issue with some groups of bacteria (11, 29), it is not generally correct. FabI inhibitors are effective against Listeria monocytogenes (31), Chlamydia trachomatis (32, 33), Neisseria gonorrhoeae (6), and S. aureus (11) in the presence of exogenous FAs. S. aureus acetyl-CoA carboxylase mutants (Δ*accD*) are grown in the laboratory in medium supplemented with branched-chain FAs (11) but fail to establish a blood infection in mice (34), suggesting that host FAs alone cannot support growth. Others conclude that S. aureus can bypass FASII inhibition based on the genotypes/phenotypes of strains isolated from infected individuals obtained by selecting for drug resistance on medium containing FA supplements (35–37). However, clones arising from selection outside the host cannot be used to infer the phenotypes of the parent bacteria, because one cannot determine whether the derived mutants arose *in vivo* or from the *in vitro* selection that occurred after the strains were recovered from the host (2).

Here, we provide a direct answer to the question of how S. aureus uses host FAs by determining the membrane phospholipid molecular species composition of S. aureus and its mutant derivatives at the thigh infection site. These data show that S. aureus uses host FAs that are activated by the FA kinase system to construct its membrane. FakB1 is the FA binding protein responsible for saturated FA activation, and FakB2 is the binding protein that functions in the activation of monounsaturated FAs. Inactivation of host FA utilization did not impact the virulence of S. aureus in the thigh model. An S. aureus FA auxotroph (Δ*accD* mutant) did not proliferate in the thigh model, and a partial FA auxotroph (Δ*plsX* mutant) was attenuated. The pattern of molecular species in the thigh model is recapitulated in the laboratory using medium containing FAs and deficient in isoleucine. The data highlight the central importance of 15:0 derived from branched-chain amino acids in S. aureus membrane phospholipid synthesis that cannot be obtained from the host.

## RESULTS

### Growth of S. aureus FA kinase mutant strains in the thigh infection model.

**FIG 2:**
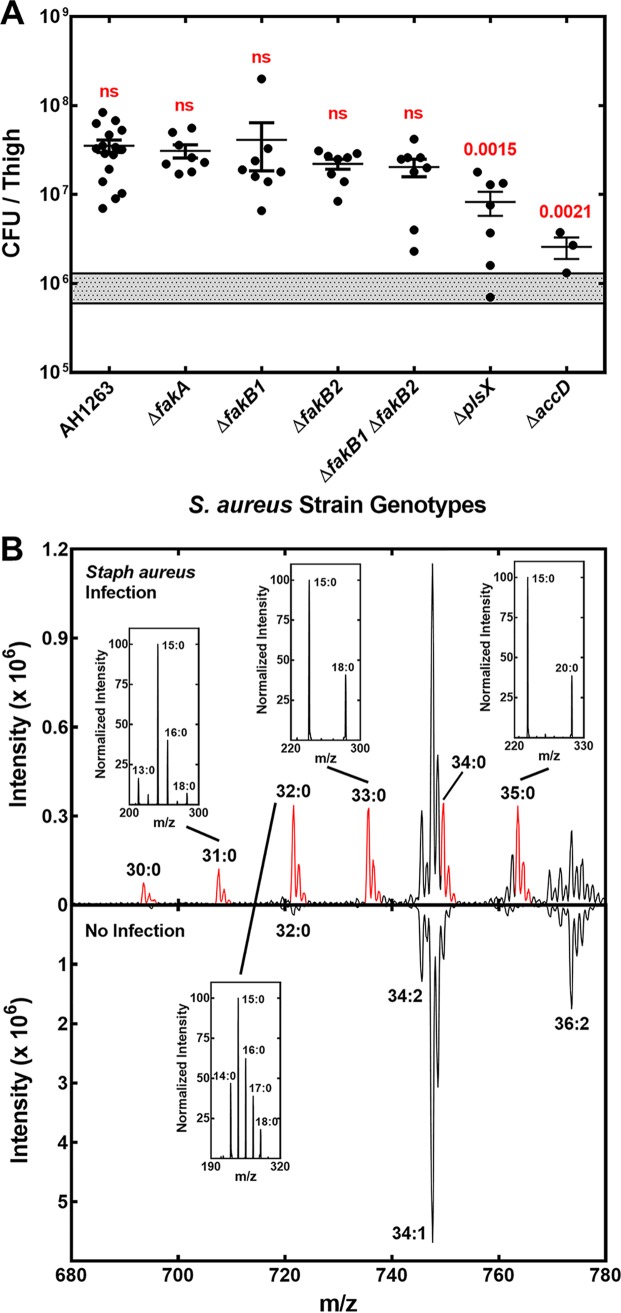
Bacterial load and detection of S. aureus PG molecular species in the neutropenic thigh model. (A) Enumeration of the bacteria recovered from the infected thighs. The thighs were infected with the indicated S. aureus strains; 24 h later, the thighs were excised, and the number of colonies present was determined by plating serial dilutions. Groups were compared using the Student's *t* test using GraphPad software, and the *P* values are shown in red. The shaded bar represents the initial inoculum. (B) A reflection plot of the PG molecular species detected in the infected and uninfected thighs. Thigh lipids were extracted, and separated into phospholipid classes by liquid chromatography, and the total negative ion scan of the peak containing PG was determined by mass spectrometry. (Top) Representative example showing the data obtained from a thigh infected with S. aureus strain JLB2 (Δ*fakA*). (Bottom) Representative example of the total negative ion current in the PG peak detected in an uninfected thigh. The new PG molecular species arising in the S. aureus infected thigh are highlighted in red. (Insets) Examples of how mass spectrometry was used to determine the FA composition of the new peaks in the infected tissue. 31:0 PG consisted of predominately 16:0 and 15:0 PGs with significantly smaller amounts of 18:0 and 13:0 PGs. 32:0 PG contained 16:0 from host PG and was a mixture of 17:0 and 15:0 PGs and 18:0 and 14:0 PGs from S. aureus. 33:0 PG was 18:0 and 15:0 PGs, and 35:0 PG was 20:0 and 15:0 PGs from S. aureus.

A panel of isogenic S. aureus strains were inoculated into the thighs of neutropenic mice (38–43); the infected thighs were excised, and the bacterial cells were enumerated 24 h later (Fig. 2A). There were no differences in the bacterial loads in the thighs infected with the various S. aureus strains with two notable exceptions. The most compromised strain was strain PDJ69 (Δ*accD* mutant). Strain PDJ69 (Δ*accD*) lacks acetyl-CoA carboxylase activity and *de novo* FA synthesis, meaning that it is a FA auxotroph (11). The Δ*accD* mutant is maintained in the lab by growth in medium supplemented with a mixture of 15- and 17-carbon branched-chain FAs (34). The inability of strain PDJ69 (Δ*accD*) to thrive illustrates that FA auxotrophs cannot utilize host FAs to support normal proliferation in the neutropenic thigh model. The second strain with a diminished capacity to thrive was strain PDJ70 (Δ*plsX* mutant) that lacks the ability to convert acyl-ACP arising from FASII to acyl-PO_4_ required by PlsY to initiate phospholipid synthesis by transferring the FA chain to glycerol phosphate (Fig. 1). Thus, Δ*plsX* strains require acyl-PO_4_ from exogenous FAs for incorporation into the 1 position and utilize *de novo* FASII to produce 15:0-ACP that is introduced into the 2 position by PlsC (44) (Fig. 1). In the laboratory, growth is supported by a number of mammalian FAs, and when strain PDJ70 (Δ*plsX*) is grown with oleate, a single PG molecular species (18:1/15:0) predominates (44). These data show that S. aureus strains that are either total (Δ*accD*) or partial (Δ*plsX*) FA auxotrophs were less fit to survive in the thigh infection model. Conversely, there was no growth defect in strains completely or partially lacking the ability to acquire host FAs, as illustrated by the bacterial titers recovered from the panel of mutants in the FA kinase system (Fig. 2A).

### S. aureus PG molecular species at the infection site.

PG was selected to determine the membrane phospholipid molecular species composition of S. aureus at the infection site, because PG is the most abundant phospholipid in S. aureus and is a relatively minor mammalian phospholipid class. After aliquots of the homogenized infected tissue sample were removed to enumerate the bacteria, the lipids were extracted, separated, and analyzed by liquid chromatography-mass spectrometry (LC-MS) as outlined in Materials and Methods. PG eluted at 4.3 min in the gradient, and the total negative ion scan of molecules in the PG peak was determined. As an example, the PG molecular species recovered from a thigh infected with strain JBL2 (Δ*fakA* mutant) is illustrated with the PG molecular species from an uninfected thigh shown in the reflection plot (Fig. 2B). Host tissue contains even-numbered saturated, monounsaturated, or polyunsaturated FAs, whereas S. aureus produces only saturated FAs. Many of the S. aureus FAs are odd numbered due to the formation of branched-chain FAs from Ile and Leu amino acid precursors. Thus, there were no odd-number PG molecular species detected in the uninfected thigh (Fig. 2B). New disaturated and odd-numbered PG molecular species appeared in the infected samples that corresponded to the PG molecular species typically detected in S. aureus grown in the laboratory (Fig. 2B, red highlights). The dominant host PG molecular species was 34:1 (16:0/18:1 PG). There were smaller quantities of 34:2 (16:0/18:2), 36:2 (18:1/18:1), and 32:0 (16:0/16:0) host PG molecular species present. Almost all of the S. aureus PG molecular species contained 15:0 in the 2 position when grown in the laboratory (11, 44), and the new PG molecular species detected in the infected thigh were fragmented to determine the FA combinations present. The positional distribution of FAs is also revealed by the fragmentation pattern of individual phospholipid molecular species (45–47). The new PG molecular species at the infection site contained predominately 15:0 as one component, and the intensity of the 15:0 signal was always higher (>2) than that of the paired FA, which is diagnostic for 15:0 occupying the 2 position (11, 44). The 30:0 peak contained only 15:0 (not shown), and the 31:0 peak was predominately 16:0/15:0 with a smaller contribution from 18:0/13:0 (Fig. 2B, insets). The 33:0 peak was 18:0/15:0 and the 35:0 peak was 20:0/15:0 (Fig. 2B, insets). The 32:0 PG peak was contaminated with 16:0/16:0 PG from the host (Fig. 2B), but the increased levels in the infected tissue arose from an increase in 17:0/15:0 PG with a lesser amount of 18:0/14:0 PG (Fig. 2B, insets). The bacterial 34:0 PG (19:0/15:0) species overlaps with isotope peaks derived from the host 34:1 PG peak (Fig. 2B). One caveat to this analysis is that if any of the S. aureus strains actually produced a 34:1 PG molecular species, we would be not able to detect it due to the overlap with the mammalian 34:1 PG. With this small caveat, these results showed that 15:0 was the major 2 position FA in the new PG molecular species that appeared in the S. aureus-infected thighs.

Column chromatography of the lipid extract was used to enrich for PGs and remove any contaminating neutral or phospholipids. These samples were analyzed by direct injection mass spectrometry using a scan for the loss of *m/z* 241 that corresponds to a 15:0 FA to image the S. aureus-derived PG molecular species. Mammalian phosphatidylinositol (PI) copurified with PGs under these column conditions and PI molecular species were also detected in the scan because the loss of *m/z *241, a fragment containing the inositol headgroup, also detects all PI molecular species. This made PI an internal marker, and PI molecular species were the only components detected in the uninfected thigh (see Fig. S1 in the supplemental material, reflection panel). The major PI molecular species was 18:0/18:2 PI. The *m/z *241 scan of a thigh infected with wild-type S. aureus strain AH1263 showed a collection of new peaks that were absent from the uninfected thigh (Fig. S1, red highlights). These peaks represented the PG molecular species containing 15:0 produced by S. aureus at the infection site.

10.1128/mBio.00920-20.1 FIG S1 Reflection plot highlighting the S. aureus PG molecular species containing 15:0 FA in the infected thigh. The thigh lipid extract was enriched for PG and phosphatidylinositol (PI) using solid-phase extraction, and the PG molecular species containing 15:0 were detected using mass spectrometry by scanning for parent ions that lose *m/z *241 corresponding to the loss of a 15:0 FA. Mouse PI was also detected because it copurified with PG and its fragmentation also gives a loss of *m/z *241 due to the removal of the inositol headgroup. (Top) *m/z *241 scan from a thigh infected with strain AH1263 (wild type). (Bottom) *m/z *241 scan from an uninfected thigh. The new PG molecular species peaks that appear in the S. aureus infected thigh are highlighted in red, and the mammalian PI molecular species are shown in blue. Download FIG S1, PDF file, 0.1 MB. Copyright © 2020 Frank et al. 2020 Frank et al. This content is distributed under the terms of the Creative Commons Attribution 4.0 International license.

**FIG 3:**
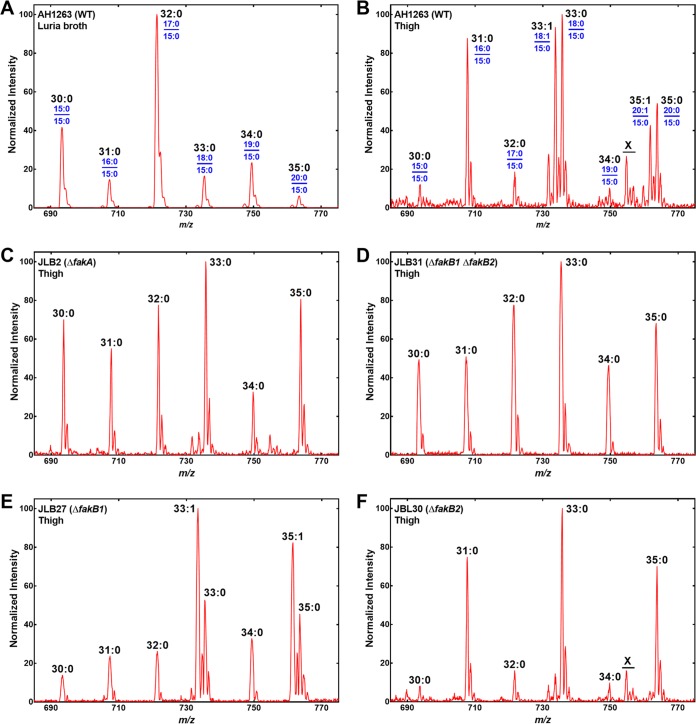
Role of FA kinase in S. aureus PG molecular species composition in the infected thigh. The thigh lipid samples were separated by solid-phase extraction, and the PG molecular species containing 15:0 were detected using mass spectrometry by scanning for parent ions that lose *m/z *241 corresponding to the loss of a 15:0 FA. The identities of the FAs in the 1 and 2 positions (blue) are shown in panels A and B for each molecular species (black). (A) Wild-type strain AH1263 grown in rich laboratory medium. (B) Strain AH1263 PG recovered from an infected thigh. (C) Strain JLB2 (Δ*fakA*) PG recovered from an infected thigh. (D) Strain JLB31 (Δ*fakB1* Δ*fakB2*) PG recovered from an infected thigh. (E) Strain JLB27 (Δ*fakB1*) PG recovered from an infected thigh. (F) Strain JLB30 (Δ*fakB2*) PG recovered from an infected thigh. Representative spectra are shown, and the triplicate results are tabulated in Table S1 in the supplemental material. The peak labeled “X” is not a PG molecular species.

The typical PG molecular species of wild-type S. aureus strain AH1263 grown in the laboratory on rich medium has a preponderance of molecules containing branched-chain FAs (Fig. 3A) (11, 44). The most abundant molecular species was 32:0 PG (17:0/15:0) (*m/z *721), followed by 34:0 PG (19:0/15:0) and 30:0 PG (15:0/15:0) molecular species. The three PG species in lowest abundance were odd-numbered molecular species that contained an even-numbered FA (16:0, 18:0, or 20:0) paired with 15:0 (Fig. 3A). Strain AH1263 growing in the infected thigh exhibited a distinctly different distribution of PG molecular species (Fig. 3B). Most notable was the appearance of 33:1 PG (18:1/15:0) (*m/z *733) and 35:1 PG (20:1/15:0) (*m/z *761), indicating the utilization of host 18:1 for phospholipid synthesis (Fig. 3B). The 20:1 in the 35:1 PG molecular species is a minor host FA and may instead arise from the elongation of 18:1 by FASII (Fig. 1A), as is observed in the laboratory when S. aureus is grown with an 18:1 supplement (11). Another obvious feature was that PG molecular species containing two branched-chain FAs were less abundant in S. aureus at the infection site. Instead, PG molecular species containing 15:0 paired with 16-, 18-, or 20-carbon FAs were significantly more abundant in S. aureus grown in the thigh model.

10.1128/mBio.00920-20.3 TABLE S1 Quantification of the PG molecular species distribution found in the PG of strains grown in the thigh model. Multiple spectra were obtained using the methods outlined in Fig. 3; the percentage of each peak was calculated and the percent distribution determined. Download Table S1, PDF file, 0.1 MB. Copyright © 2020 Frank et al. 2020 Frank et al. This content is distributed under the terms of the Creative Commons Attribution 4.0 International license.

A series of mutants deficient in the individual protein components of the FA kinase system were analyzed in the thigh model to determine their role(s) in host FA utilization for membrane phospholipid synthesis. Strain JLB2 (Δ*fakA* mutant) lacks the kinase domain protein and cannot incorporate any exogenous FAs (8). The PG molecular species of this strain lacked the 33:1 and 35:1 PG peaks arising from host 18:1 incorporation and had a higher proportion of molecular species containing an even-chain saturated FA paired with 15:0 (Fig. 3C) than the wild-type strain grown in the lab on rich medium (Fig. 3A). Strain JLB31 (Δ*fakB1* Δ*fakB2*) is missing both FakB components of FA kinase and, like the Δ*fakA* strain, cannot activate or incorporate FA (8). The PG molecular species profile of strain JLB31 (Fig. 3D) was similar to that of the Δ*fakA* strain (Fig. 3C), as expected from their inability to activate exogenous FA. Strain JBL27 (Δ*fakB1* mutant) lacked the FA binding protein specific for saturated FAs but has a functional FakB2 that is selective for 18:1 (8, 10). The Δ*fakB1* strain exhibited an increase in 18:1/15:0 and 20:1/15:0 PG at the expense of molecular species containing even-chain saturated FAs, notably, the 31:0 PG (16:0/15:0) peak (Fig. 3E). Strain JLB30 (Δ*fakB2* mutant) lacks the FA binding protein specific for monounsaturated FAs (8–10). PG molecular species containing 18:1 and 20:1 were missing, and there was an increase in molecular species containing even-chain saturated FAs (Fig. 3F). These data show that S. aureus utilizes host FAs by the FA kinase system and that the selectivity of the FakB components determined the extent of saturated and monounsaturated FA incorporation into PG molecular species. The percent contributions of each PG molecular species to the total PG ion current were calculated, and the averages are listed in Table S1 to summarize the impact of mutations in the FA kinase system on S. aureus PG molecular species composition at the infection site. These data present a comprehensive picture of the role of the FA kinase genes in the acquisition of host FAs for membrane biogenesis at the infection site.

### PG molecular species in S. aureus FA auxotrophs.

Strain PDJ69 (Δ*accD*) cannot synthesize FA, and there was insufficient bacterial material recovered from thighs infected with this strain for subsequent lipidomic analysis due to its marked growth defect (Fig. 2A). Strain PDJ70 (Δ*plsX*) is a partial FA auxotroph that incorporates an exogenous FA into the 1 position of PG and 15:0 into the 2 position (44). Recovery of the Δ*plsX* strain was lower in the thigh model than that of the wild type (Fig. 2A), but there was enough material to image the PG molecular species. This strain cannot elongate exogenous FAs, because PlsX is the link between FA kinase and FASII (Fig. 1). When grown in the laboratory with an 18:1 supplement, the only PG molecular species was *m/z* 733 corresponding to 33:1 PG (18:1/15:0), and there was no elongation of 18:1 to 20:1 (see Fig. S2A). In the Δ*plsX* strain recovered from the thigh model, the two most abundant PG molecular species detected were 31:0 PG (16:0/15:0) and 33:1 PG (18:1/15:0), with a smaller amount of 33:2 PG (18:2/15:0) also present (Fig. S2B). There was no evidence for molecular species containing 18:0, 20:0, or 20:1, consistent with the inability of Δ*plsX* strains to elongate FAs. These data indicated that the major FA species available in the thigh were 16:0 and 18:1 and that PG molecular species containing the longer-chain FA in strains containing an active PlsX arose from the elongation of these two host-derived FA nutrients.

10.1128/mBio.00920-20.2 FIG S2 Host FA utilization by strain PDJ70 (Δ*plsX*). The identities of the FAs in the 1 and 2 positions (blue) are shown for each molecular species (black). (A) A representative mass spectrum of the PG molecular species of strain PDJ70 (Δ*plsX*) used to inoculate the thigh grown in tryptone broth supplemented with 500 μM 18:1. (B) A representative mass spectrum of PG molecular species from strain PDJ70 (Δ*plsX*) recovered from the thigh infection site. The PG fraction was isolated, and the mass spectrum of the sample was generated using an *m/z *241 scan to detect PG molecular species containing 15:0 (see Fig. S1). The peak labeled “X” is not a PG molecular species. Download FIG S2, PDF file, 0.2 MB. Copyright © 2020 Frank et al. 2020 Frank et al. This content is distributed under the terms of the Creative Commons Attribution 4.0 International license.

### Factors affecting PG structure.

**FIG 4:**
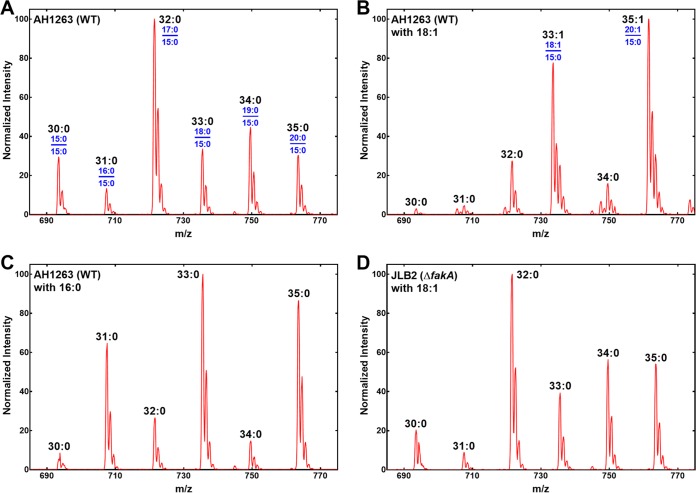
Impact of exogenous FAs on PG molecular species. Strains were grown in defined media (described in Materials and Methods) plus 0.1% Brij-58 and with or without 500 μM FAs. The identities of the FAs in the 1 and 2-positions (blue) are shown in panels A and B for each molecular species (black). (A) Strain AH1263 grown in defined medium. (B) PG molecular species of strain AH1263 grown in defined medium broth containing 500 μM 18:1. (C) Strain AH1263 grown in defined medium supplemented with 500 μM 16:0. (D) Strain JLB2 (Δ*fakA*) grown in defined medium containing 500 μM 18:1.

The comparison of the PG molecular species in strain AH1263 in rich medium (Fig. 3A) compared to those in the thigh (Fig. 3B) shows that even-numbered PG molecular species containing two branched-chain FAs were replaced by odd-numbered PG species composed of one even-numbered FA paired with 15:0. This effect was modeled in the laboratory by growing strains AH1263 and JLB2 (Δ*fakA*) in Luria broth containing 500 μM 16:0 or 18:1. In the wild-type strain AH1263, growth with extracellular 18:1 converted the normal distribution of PG molecular species dominated by even-numbered FAs (Fig. 4A) to a simplified pattern containing primarily 33:1 (18:1/15:0) and 35:1 (20:1/15:0) PG (Fig. 4B). Similarly, the addition of 16:0 suppressed the synthesis of all FAs except 15:0, creating a PG molecular species composition containing predominately 31:0 (16:0/15:0), 33:0 (18:0/15:0), and 35:0 (20:0/15:0) PGs (Fig. 4C). Experiments feeding the strain with [*d*_3_]16:0 to introduce a mass tag showed that the 18:0 and 20:0 FAs arose from elongation of 16:0 (not shown). Strain JLB2 (Δ*fakA*) cannot activate exogenous FA, and the growth of this strain with exogenous 18:1 did not impact FASII product distribution (Fig. 4D), resulting in a PG molecular species composition that closely matched that in the absence of extracellular FAs (Fig. 4A). These data showed that exogenous FAs suppressed *de novo* FA formation, except for 15:0. The 15:0 ACP produced by FASII was used to acylate the 2 position at the PlsC step. These data revealed that the utilization of host FA coupled with the suppression of FASII (except for 15:0) contributed to the observed membrane composition of S. aureus grown in the thigh.

**FIG 5:**
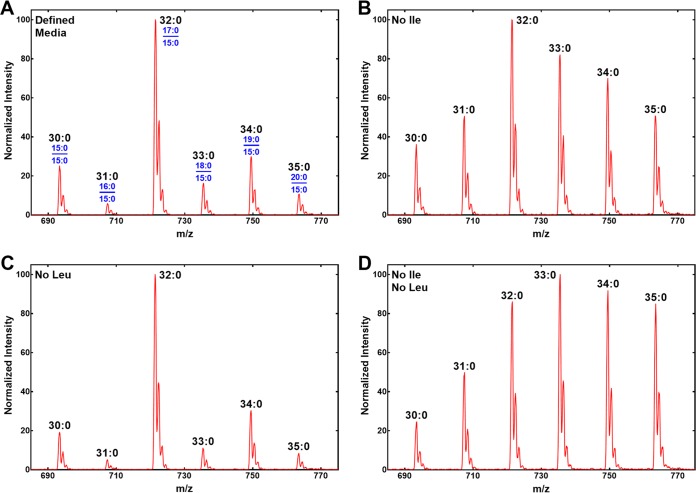
Impact of exogenous Ile on PG molecular species of wild-type strain AH1263. The identities of the FAs in the 1 and 2 positions (blue) are shown in panel A for each PG molecular species (black). (A) PG molecular species of strain AH1263 grown in defined medium as described in Materials and Methods containing 50 μg/ml Ile and Leu. (B) PG molecular species of strain AH1263 grown in defined medium lacking Ile. (C) PG molecular species of strain AH1263 grown in defined medium lacking Leu. (D) PG molecular species of strain AH1263 grown in defined medium lacking both Ile and Leu.

S. aureus strains that were incapable of activating host FAs (Δ*fakA* or Δ*fakB1* Δ*fakB2* mutants) also exhibited an increase in odd-numbered PG molecular species in the thigh infection site (Fig. 3C and D) compared to growth in rich medium (Fig. 3A). We performed a series of experiments to determine if the reduced amount of branched-chain FAs could be attributed to the availability of extracellular amino acid precursors, Ile and Leu. These two amino acids are metabolized by transamination (IlvE) followed by branched-chain ketoacid dehydrogenase to form the respective isomeric C_5_-CoAs, which are used by FabH to initiate branched-chain FA synthesis. Defined media were prepared that contained combinations of Leu and Ile. Growth of strain AH1263 in the defined medium containing 50 μg/ml Ile and Leu produced a PG molecular species profile with an abundance of even-numbered molecular species containing branched-chain FAs (Fig. 5A), reflecting that of the strain grown in Luria broth (Fig. 4A). The removal of Ile from the medium resulted in a marked increase in odd-numbered PG molecular species (Fig. 5B) that closely resembled the compositions of the FA kinase null mutants in the thigh (Fig. 3C and D). Removal of Leu from the medium had no effect on the PG molecular species composition (Fig. 5C). The PG species when both Ile and Leu were absent when the medium was the same as when only Ile was removed (Fig. 5D). These data showed that the difference between the PG molecular species of the Δ*fakA* strain in the thigh and that in rich medium was mimicked in the laboratory by growth in Ile-deficient medium. This suggests a limited availability of Ile at the infection site.

## DISCUSSION

This work defines the phospholipid molecular species composition of S. aureus at the thigh infection site and the role of the FA kinase system in the utilization of host FAs during an infection for membrane biogenesis. The FA kinase system is responsible for the activation of extracellular FAs, and the acyl-PO_4_ intermediates are either used by PlsY or converted to acyl-ACP by PlsX (Fig. 1). The acyl-ACP has two possible fates: PlsC or FASII (Fig. 1). Because PlsC is selective for 15:0 ACP (11, 12), few mammalian longer-chain FAs, such as 16:0 and 18:1, that are converted to acyl-ACP are utilized by PlsC to acylate the 2 position. Rather, acyl-ACPs derived from host FAs are substrates for FabF and enter the elongation cycle of FASII (Fig. 1). The elongated acyl-ACP is again converted to acyl-PO_4_ by PlsX and used to acylate the 1 position at the PlsY step (Fig. 1). S. aureus FASII fails to elongate 20-carbon acyl-ACP, putting an upper limit on chain length. Knocking out the monounsaturated FA binding protein (FakB2) that prefers to bind 18:1 results in the elimination of monounsaturated PG molecular species coupled with an increase in PG molecular species containing saturated even-chain FAs. This result verifies the key role of FakB2 in acquiring host 18:1 (Fig. 1). The elimination of the saturated FA binding protein (FakB1) that prefers to bind 16:0 results in an increase in monounsaturated PG molecular species coupled with a reduction in saturated PG species, verifying the role of FakB1 in saturated FA activation (Fig. 1). Although the individual spectra cannot distinguish between even-chain saturated FAs produced by S. aureus FASII or obtained from the host, the PG molecular species compositions of the wild-type compared to those of the Δ*fakA*, Δ*fakB1*, and Δ*fakB2* mutant strains show that S. aureus actively scavenges host saturated FAs. We conclude that 16:0 and 18:1 are the primary FA utilized by S. aureus in the thigh based on the results using strain PDJ70 (Δ*plsX*). This strain is an FA auxotroph and must obtain a FA for incorporation into the 1 position from the host. The abundance of 31:0 and 33:1 PG molecular species in the PG molecular species of the Δ*plsX* strain recovered from the thigh indicates that 16:0 and 18:1 are the primary host FAs activated by S. aureus at the infection site. This conclusion is also consistent with the established substrate selectivity of FakB1 for 16:0 and FakB2 for 18:1 (8–10), meaning that the PG molecular species containing 18:0, 20:0, and 20:1 arise from the elongation of 16:0 or 18:1. Although the S. aureus FA kinase system is designed to scavenge two of the most abundant host FAs, strains lacking an FA kinase (Δ*fakA* or Δ*fakB1* Δ*fakB2* mutants) still proliferate to the same extent in the thigh as the wild type, illustrating that the acquisition of host FAs does not significantly contribute to fitness in the thigh model.

Although FA auxotrophs are easily grown in the laboratory with exogenous FA supplements, they exhibit a fitness defect in the thigh infection model. Strain PDJ69 (Δ*accD*) is a complete FA auxotroph and did not proliferate in the thigh model. This result is consistent with the earlier observation that S. aureus strain JP103 (Δ*accD* mutant) was unable to establish bloodstream infection in mice (34). The Δ*accD* strain does not grow continuously in laboratory cultures supplemented with mammalian even-chain FAs, whereas normal growth in the laboratory is supported by supplying a mixture of 15:0 and 17:0 FAs (34). This result emphasizes the importance of branched-chain FAs, which are not available in the host, to S. aureus physiology. Strain PDJ70 (Δ*plsX*) is also an FA auxotroph but differs from the Δ*accD* strain in that exogenous FAs are only required for acylation of the 1 position by PlsY while *de novo* biosynthesis provides the 15:0 for PlsC to acylate the 2 position (Fig. 1) (44). Mammalian FAs, such as 18:1, fully support the growth of Δ*plsX* strains in the lab (44). The Δ*plsX* strain nonetheless exhibits a proliferation defect in the thigh model (Fig. 2A), indicating that exogenous FA availability may be a limiting nutrient for this partial FA auxotroph. These data illustrating the dependence of S. aureus on 15:0 leads to a deeper understanding of why the FabI-directed drug, AFN-1252 (afabicin), is an effective antibiotic (23, 24, 27, 28). Shutting off FASII prevents the formation of branched-chain FAs that are not available from the host but are essential components of the S. aureus membrane at the infection site.

Our results indicate that the availability of branched-chain amino acids at the infection site also impacts membrane phospholipid structure. S. aureus is typically grown in rich laboratory medium containing abundant Ile and Leu, leading to the formation of PG molecular species with a high proportion of branched-chain FAs. However, if the strain is grown in medium containing host FAs, the synthesis of FAs other than 15:0 is suppressed, leading to a PG molecular species composition that has the host FA (or its elongation product) paired with 15:0. The PG molecular species of strains that cannot utilize host FAs (Δ*fakA* or Δ*fakB1* Δ*fakB2* mutant) also contain a higher proportion of even-chain FAs. This compositional change is recapitulated in the laboratory by growth of S. aureus in medium with little or no Ile. When Ile is limiting in the medium, the cells compensate by increasing the synthesis of even-number FAs to pair with 15:0. These data lead to the conclusion that the thigh infection site is a source of 16:0 and 18:1 FAs that are not normally provided in laboratory growth media and is relatively deficient in Ile compared to laboratory growth media. Low extracellular Ile is thought to be common at infection sites, and this is known to trigger the expression of many virulence genes through its binding to the global repressor, CodY (13). The biochemical mechanisms used by S. aureus to regulate the types of FAs produced by FASII in response to extracellular FA and branched-chain amino acids remain to be elucidated.

## MATERIALS AND METHODS

### Materials.

Components of defined media were from Millipore Sigma or Thermo Fisher Scientific. Oleate, palmitate, fatty acid free-bovine serum albumin, and Discovery DSC-NH2 solid-phase extraction columns were from Millipore Sigma. The 15:0 and 17:0 FAs were from Larodan. The Acquity UPLC BEH HILIC, 1.7-μm 2.1-mm by 150-mm column was from Waters. All solvents for mass spectrometry were high-performance liquid chromatography (HPLC) grade or better.

### Bacterial strains.

The strains used in this study are listed in Table S2 in the supplemental material. Strain PDJ69 (Δ*accD*) was constructed from the strain AH1263 by allelic replacement. Briefly, ∼1,000 bp from either side of *accD* were amplified by PCR using primers AccD-Up-F, AccD-Up-R, AccD-Dn-F, and AccD-Dn-R. The PCR products were moved into pJB38 by Gibson assembly (New England BioLabs) and transformed into AH1263 by electroporation, and the knockout was generated as previously described (48, 49). FA auxotroph knockouts were selected on tryptic soy agar plates supplemented with 10 mg/ml FA-free bovine serum albumin, 15:0 (333 μM), and 17:0 (167 μM) FA. The Δ*accD* knockout strain PDJ69 was confirmed by PCR using primers AccD-Ex-F and AccD-Ex-R, with a knockout product size of 2,075 bp compared to the wild-type product size of 2,866 bp.

10.1128/mBio.00920-20.4 TABLE S2 Bacterial strains. 51, B. R. Boles, M. Thoendel, A. J. Roth, and A. R. Horswill AR, PLoS One 5:e10146, 2010, https://doi.org/10.1371/journal.pone.0010146. Download Table S2, PDF file, 0.1 MB. Copyright © 2020 Frank et al. 2020 Frank et al. This content is distributed under the terms of the Creative Commons Attribution 4.0 International license.

Strain PDJ70 (Δ*plsX*) was constructed from strain AH1263 by the insertion of a group II intron 366 bp into the *plsX* gene using the TargeTron gene knockout system (Sigma-Aldrich) as in our previous study (44). Knockouts were selected on plates containing 10 mg/ml FA-free bovine serum albumin and 1 mM 18:1 FA. Genotyping was performed using a multiplex PCR containing a primer specific for the intron (5′-CGAAATTAGAAACTTGCGTTCAGTAAAC) and two *plsX* gene-specific primers (280F, 5′-CAGCAGGTAATACTGGTGCTTTAATGTCAG and 789R, 5′-ATCTTTCTTCAATATTGCACCTGC). The knockout allele gave a 305-bp product compared to the wild-type *plsX* product size of 509 bp.

### Growth media.

Defined medium consisted of M9 salts, 1 mM MgSO_4_, 0.1 mM CaCl_2_, 15 μM vitamin B_1_, 32 μM vitamin B3, 0.4% glucose, 0.1 mg/liter biotin, 2 mg/liter pantothenic acid, 10 μM FeCl_2_, 6 mg/liter citrate, 10 mg/liter MnCl_2_, 4 mg/liter l-tryptophan, and 0.1 mg/liter lipoic acid, and the amino acid concentrations were the same as in RPMI 1640 medium (Sigma-Aldrich). Strain AH1263 was grown in Luria broth, strain PDJ70 (Δ*plsX*) was grown in Luria broth plus 10 mg/ml fatty acid free-bovine serum albumin plus 1 mM 18:1, and strain (Δ*accD*) was grown in the same medium supplemented with 1 mM 15:0 and 1 mM 17:0.

### Isoleucine/leucine growth experiments.

Overnight culture of strain AH1263 was in defined medium that contained Ile and Leu. Overnight cells were collected by centrifugation at 4,000 × *g* for 10 min at room temperature, were washed with defined medium lacking Ile and Leu, and were collected by centrifugation. The cell pellet was resuspended in defined medium lacking Ile and Leu. These cells were used to inoculate defined medium that contained the following: 50 μg/ml Ile and 50 μg/ml Leu; 50 μg/ml Ile and no Leu; 50 μg/ml Leu and no Ile; or no Ile or Leu. Cultures were grown for 8 h, cells were collected, and lipids were extracted for mass spectrometry.

### Culturing with fatty acids.

Cultures of strains AH1263 or JBL2 (Δ*fakA*) were grown at 37°C in defined medium plus 0.1% Brij-58 to an optical density at 600 nm (OD_600_) of 0.2. Cell cultures were treated with either 500 μM oleate, 500 μM palmitate, or dimethyl sulfoxide (DMSO; vehicle control) and were incubated for 5 h. Cells were harvested by centrifugation, and the lipids were extracted for mass spectrometry.

### Lipid extraction.

Thigh homogenates and bacterial cells were extracted using the method of Bligh and Dyer (50). Four thighs were combined to obtain the material for one lipid analysis. The thigh lipid extract was enriched for PG plus PI using solid-phase extraction. In brief, the solid-phase column (Discovery DSC-NH2, 500 mg) was conditioned with 8 ml of hexane, and lipid extract was added. Nonpolar lipids were eluted with 6 ml of 2:1 (vol/vol) chloroform/isopropanol; fatty acids were eluted with 6 ml of ether plus 2% acetic acid; phosphatidylcholines (PC) and phosphatidylethanolamine (PE) were eluted with 6 ml of methanol; and PG and PI were eluted with 6 ml of chloroform/methanol/0.8 M sodium acetate (60:30:4.5 [vol/vol/vol]).

### Thigh infection model.

The neutropenic thigh model is a standard approach that enables reproducible replication of S. aureus following inoculation to evaluate bacterial pathogens, including S. aureus (38–43). The cyclophosphamide regimen consistently induced profound neutropenia (<10 neutrophils/mm^3^). A minimum of five 8-week-old BALB/c female mice (Jackson Laboratory) per group were treated with a cyclophosphamide regimen previously optimized (not shown) to consistently induce neutropenia (<10 neutrophils/mm^3^) (43). Cyclophosphamide (150 mg/kg) was intraperitoneally (i.p.) injected day −5 prior to infection followed by another cyclophosphamide (100 mg/kg) i.p. injection day −2 prior to infection. Bacteria were grown in their respective media overnight at 37°C and back diluted in fresh medium and allowed to grow to an *A*_600_ of 0.4. Bacteria were washed twice with sterile phosphate-buffered saline (PBS) and suspended in fresh PBS to obtain an inoculum of 10^6^ CFU/50 μl. Bacteria were introduced via intramuscular administration of 10^6^ (50 μl) wild-type S. aureus (AH1263) or the respective mutants in PBS or 50 μl PBS alone (negative control). Bacteria were enumerated to ensure that the proper number of bacteria was used in infection doses. At 24 h postinfection, total thigh muscles were harvested in 500 μl ice-cold PBS. The thigh muscles were then homogenized followed by additionally adding 500 μl PBS. Homogenate samples were serially diluted and plated on mannitol salt agar selection plates with or without the addition of bovine serum albumin-FA for FA autotrophs (Δ*plsX* and Δ*accD*) to determine bacterial burden.

### Mass spectrometry.

Lipid extracts were resuspended in chloroform/methanol (1:1). PG was analyzed using a Shimadzu Prominence UFLC attached to a QTrap 4500 equipped with a Turbo V ion source (Sciex). Samples were injected onto an Acquity UPLC BEH HILIC, 1.7-μm 2.1-mm by 150-mm column (Waters) at 45°C with a flow rate of 0.2 ml/min. Solvent A was acetonitrile, and solvent B was 15 mM ammonium formate, pH 3. The HPLC program was the following: starting solvent mixture of 96% A/4% B; 0 to 2 min, isocratic with 4% B; 2 to 20 min, linear gradient to 80% B; 20 to 23 min, isocratic with 80% B; 23 to 25 min, linear gradient to 4% B; 25 to 30 min, isocratic with 4% B. The QTrap 4500 was operated in the Q1 negative mode. The ion source parameters for Q1 were as follows: ion spray voltage, −4,500 V; curtain gas, 25 lb/in^2^; temperature, 350°C; ion source gas 1, 40 lb/in^2^; ion source gas 2, 60 lb/in^2^; and declustering potential, −40 V. The system was controlled by the Analyst software (Sciex). The sum of the areas under each peak in the mass spectra were calculated, and the percentage of each molecular species present was calculated with LipidView software (Sciex).

Lipid extracts from the thigh were analyzed by direct injection into a QTrap 4500 equipped with a Turbo V ion source (Sciex). The instrument was operated in precursor negative mode to detect PGs that contained a 15:0 fatty acid and PI. The ion source parameters were as follows: precursor *m/z*, 241; ion spray voltage, −4,500 V; curtain gas, 15 lb/in^2^; temperature, 300°C; ion source gas 1, 15 lb/in^2^; ion source gas 2, 20 lb/in^2^; declustering potential, −35 V; and collision energy, −40 V. The system was controlled by the Analyst software (Sciex).

### Ethics statement.

All work with S. aureus described herein were conducted in accordance with protocols approved by the Institutional Biosafety Committees at St. Jude Children’s Research Hospital (SJCRH). All animal experiments were performed with prior review and approval by the St. Jude Institutional Animal Care and Use Committee (IACUC). All mice were maintained in biosafety level 2 (BSL2) facilities in accordance with IACUC protocol number 566-100442-10/16.

## References

[1] Yao J, Rock CO 2015 How bacterial pathogens eat host lipids: implications for the development of fatty acid synthesis therapeutics. J Biol Chem 290:5940–5946. doi:10.1074/jbc.R114.636241. 25648887 PMC4358231

[2] Yao J, Rock CO 2017 Exogenous fatty acid metabolism in bacteria. Biochimie 141:30–39. doi:10.1016/j.biochi.2017.06.015. 28668270 PMC5665373

[3] Black PN, DiRusso CC, Metzger AK, Heimert TL 1992 Cloning, sequencing, and expression of the *fadD* gene of *Escherichia coli* encoding acyl coenzyme A synthetase. J Biol Chem 267:25513–25520. 1460045

[4] Yao J, Rock CO 2013 Phosphatidic acid synthesis in bacteria. Biochim Biophys Acta 1831:495–502. doi:10.1016/j.bbalip.2012.08.018. 22981714 PMC3548993

[5] Jiang Y, Chan CH, Cronan JE 2006 The soluble acyl-acyl carrier protein synthetase of *Vibrio harveyi* B392 is a member of the medium chain acyl-CoA synthetase family. Biochemistry 45:10008–10019. doi:10.1021/bi060842w. 16906759

[6] Yao J, Bruhn DF, Frank MW, Lee RE, Rock CO 2016 Activation of exogenous fatty acids to acyl-acyl carrier protein cannot bypass FabI inhibition in *Neisseria*. J Biol Chem 291:171–181. doi:10.1074/jbc.M115.699462. 26567338 PMC4697154

[7] Yao J, Dodson VJ, Frank MW, Rock CO 2015 *Chlamydia trachomatis* scavenges host fatty acids for phospholipid synthesis via an acyl-acyl carrier protein synthetase. J Biol Chem 290:22163–22173. doi:10.1074/jbc.M115.671008. 26195634 PMC4571967

[8] Parsons JB, Broussard TC, Bose JL, Rosch JW, Jackson P, Subramanian C, Rock CO 2014 Identification of a two-component fatty acid kinase responsible for host fatty acid incorporation by *Staphylococcus aureus*. Proc Natl Acad Sci U S A 111:10532–10537. doi:10.1073/pnas.1408797111. 25002480 PMC4115530

[9] Broussard TC, Miller DJ, Jackson P, Nourse A, White SW, Rock CO 2016 Biochemical roles for conserved residues in the bacterial fatty acid binding protein family. J Biol Chem 291:6292–6303. doi:10.1074/jbc.M115.706820. 26774272 PMC4813577

[10] Cuypers MG, Subramanian C, Gullett JM, Frank MW, White SW, Rock CO 2019 Acyl chain selectivity and physiological roles of *Staphylococcus aureus* fatty acid binding proteins. J Biol Chem 294:38–49. doi:10.1074/jbc.RA118.006160. 30429218 PMC6322867

[11] Parsons JB, Frank MW, Subramanian C, Saenkham P, Rock CO 2011 Metabolic basis for the differential susceptibility of Gram-positive pathogens to fatty acid synthesis inhibitors. Proc Natl Acad Sci U S A 108:15378–15383. doi:10.1073/pnas.1109208108. 21876172 PMC3174620

[12] Robertson RM, Yao J, Gajewski S, Kumar G, Martin EW, Rock CO, White SW 2017 A two-helix motif positions the lysophosphatidic acid acyltransferase active site for catalysis within the membrane bilayer. Nat Struct Mol Biol 24:666–671. doi:10.1038/nsmb.3436. 28714993 PMC5616210

[13] Kaiser JC, Heinrichs DE 2018 Branching out: alterations in bacterial physiology and virulence due to branched-chain amino acid deprivation. mBio 9:e01188-18. doi:10.1128/mBio.01188-18. 30181248 PMC6123439

[14] Pohl K, Francois P, Stenz L, Schlink F, Geiger T, Herbert S, Goerke C, Schrenzel J, Wolz C 2009 CodY in *Staphylococcus aureus*: a regulatory link between metabolism and virulence gene expression. J Bacteriol 191:2953–2963. doi:10.1128/JB.01492-08. 19251851 PMC2681790

[15] Singh VK, Hattangady DS, Giotis ES, Singh AK, Chamberlain NR, Stuart MK, Wilkinson BJ 2008 Insertional inactivation of branched-chain α-keto acid dehydrogenase in *Staphylococcus aureus* leads to decreased branched-chain membrane fatty acid content and increased susceptibility to certain stresses. Appl Environ Microbiol 74:5882–5890. doi:10.1128/AEM.00882-08. 18689519 PMC2565972

[16] Singh VK, Sirobhushanam S, Ring RP, Singh S, Gatto C, Wilkinson BJ 2018 Roles of pyruvate dehydrogenase and branched-chain alpha-keto acid dehydrogenase in branched-chain membrane fatty acid levels and associated functions in *Staphylococcus aureus*. J Med Microbiol 67:570–578. doi:10.1099/jmm.0.000707. 29498620 PMC5982145

[17] Yao J, Rock CO 2018 Therapeutic targets in chlamydial fatty acid and phospholipid synthesis. Front Microbiol 9:2291. doi:10.3389/fmicb.2018.02291. 30319589 PMC6167442

[18] Yao J, Rock CO 2017 Bacterial fatty acid metabolism in modern antibiotic discovery. Biochim Biophys Acta Mol Cell Biol Lipids 1862:1300–1309. doi:10.1016/j.bbalip.2016.09.014. 27668701 PMC5364071

[19] Parsons JB, Rock CO 2011 Is bacterial fatty acid synthesis a valid target for antibacterial drug discovery? Curr Opin Microbiol 14:544–549. doi:10.1016/j.mib.2011.07.029. 21862391 PMC3193581

[20] Campbell JW, Cronan JE Jr 2001 Bacterial fatty acid biosynthesis: targets for antibacterial drug discovery. Annu Rev Microbiol 55:305–332. doi:10.1146/annurev.micro.55.1.305. 11544358

[21] Zhang Y-M, White SW, Rock CO 2006 Inhibiting bacterial fatty acid synthesis. J Biol Chem 281:17541–17544. doi:10.1074/jbc.R600004200. 16648134

[22] Karlowsky JA, Kaplan N, Hafkin B, Hoban DJ, Zhanel GG 2009 AFN-1252, a FabI inhibitor, demonstrates a *Staphylococcus*-specific spectrum of activity. Antimicrob Agents Chemother 53:3544–3548. doi:10.1128/AAC.00400-09. 19487444 PMC2715641

[23] Kaplan N, Albert M, Awrey D, Bardouniotis E, Berman J, Clarke T, Dorsey M, Hafkin B, Ramnauth J, Romanov V, Schmid MB, Thalakada R, Yethon J, Pauls HW 2012 Mode of action, *in vitro* activity, and *in vivo* efficacy of AFN-1252, a selective antistaphylococcal FabI inhibitor. Antimicrob Agents Chemother 56:5865–5874. doi:10.1128/AAC.01411-12. 22948878 PMC3486558

[24] Banevicius MA, Kaplan N, Hafkin B, Nicolau DP 2013 Pharmacokinetics, pharmacodynamics and efficacy of novel FabI inhibitor AFN-1252 against MSSA and MRSA in the murine thigh infection model. J Chemother 25:26–31. doi:10.1179/1973947812Y.0000000061. 23433441 PMC3558988

[25] Flamm RK, Rhomberg PR, Kaplan N, Jones RN, Farrell DJ 2015 Activity of Debio1452, a FabI inhibitor with potent activity against *Staphylococcus aureus* and coagulase-negative *Staphylococcus spp*., including multidrug-resistant strains. Antimicrob Agents Chemother 59:2583–2587. doi:10.1128/AAC.05119-14. 25691627 PMC4394798

[26] Yao J, Maxwell JB, Rock CO 2013 Resistance to AFN-1252 arises from missense mutations in *Staphylococcus aureus* enoyl-acyl carrier protein reductase (FabI). J Biol Chem 288:36261–36271. doi:10.1074/jbc.M113.512905. 24189061 PMC3868742

[27] Hafkin B, Kaplan N, Murphy B 2015 Efficacy and safety of AFN-1252, the first *Staphylococcus*-specific antibacterial agent, in the treatment of acute bacterial skin and skin structure infections, including those in patients with significant comorbidities. Antimicrob Agents Chemother 60:1695–1701. doi:10.1128/AAC.01741-15. 26711777 PMC4775962

[28] Menetrey A, Janin A, Pullman J, Overcash JS, Haouala A, Leylavergne F, Turbe L, Wittke F, Nicolas-Métral V 2018 Bone and joint tissue penetration of the *Staphylococcus*-selective antibiotic afabicin in patients undergoing elective hip replacement surgery. Antimicrob Agents Chemother 63:e01669-18. doi:10.1128/AAC.01669-18. PMC6395911 30559136

[29] Brinster S, Lamberet G, Staels B, Trieu-Cuot P, Gruss A, Poyart C 2009 Type II fatty acid synthesis is not a suitable antibiotic target for Gram-positive pathogens. Nature 458:83–86. doi:10.1038/nature07772. 19262672

[30] Brinster S, Lamberet G, Staels B, Trieu-Cuot P, Gruss A, Poyart C 2010 Brinster et al. reply. Nature 463:E4. doi:10.1038/nature08668. 19262672

[31] Yao J, Ericson ME, Frank MW, Rock CO 2016 Enoyl-acyl carrier protein reductase I (FabI) is essential for the intracellular growth of *Listeria monocytogenes*. Infect Immun 84:3597–3607. doi:10.1128/IAI.00647-16. 27736774 PMC5116736

[32] Yao J, Abdelrahman YM, Robertson RM, Cox JV, Belland RJ, White SW, Rock CO 2014 Type II fatty acid synthesis is essential for the replication of *Chlamydia trachomatis*. J Biol Chem 289:22365–22376. doi:10.1074/jbc.M114.584185. 24958721 PMC4139244

[33] Yao J, Cherian PT, Frank MW, Rock CO 2015 *Chlamydia trachomatis* relies on autonomous phospholipid synthesis for membrane biogenesis. J Biol Chem 290:18874–18888. doi:10.1074/jbc.M115.657148. 25995447 PMC4521007

[34] Parsons JB, Frank MW, Rosch JW, Rock CO 2013 *Staphylococcus aureus* fatty acid auxotrophs do not proliferate in mice. Antimicrob Agents Chemother 57:5729–5732. doi:10.1128/AAC.01038-13. 23979734 PMC3811263

[35] Morvan C, Halpern D, Kenanian G, Pathania A, Anba-Mondoloni J, Lamberet G, Gruss A, Gloux K 2017 The *Staphylococcus aureus* FASII bypass escape route from FASII inhibitors. Biochimie 141:40–46. doi:10.1016/j.biochi.2017.07.004. 28728970

[36] Gloux K, Guillemet M, Soler C, Morvan C, Halpern D, Pourcel C, Vu Thien H, Lamberet G, Gruss A 2017 Clinical relevance of type II fatty acid synthesis bypass in *Staphylococcus aureus*. Antimicrob Agents Chemother 61:e02515-16. doi:10.1128/AAC.02515-16. 28193654 PMC5404599

[37] Morvan C, Halpern D, Kenanian G, Hays C, Anba-Mondoloni J, Brinster S, Kennedy S, Trieu-Cuot P, Poyart C, Lamberet G, Gloux K, Gruss A 2016 Environmental fatty acids enable emergence of infectious *Staphylococcus aureus* resistant to FASII-targeted antimicrobials. Nat Commun 7:12944. doi:10.1038/ncomms12944. 27703138 PMC5059476

[38] Lepak AJ, Zhao M, Andes DR 2017 Comparative pharmacodynamics of telavancin and vancomycin in the neutropenic murine thigh and lung infection models against *Staphylococcus aureus*. Antimicrob Agents Chemother 61:e00281-17. doi:10.1128/AAC.00281-17. 28416551 PMC5487664

[39] Bulik CC, Okusanya OO, Lakota EA, Forrest A, Bhavnani SM, Hoover JL, Andes DR, Ambrose PG 2017 Pharmacokinetic-pharmacodynamic evaluation of gepotidacin against Gram-positive organisms using data from murine infection models. Antimicrob Agents Chemother 61:e00115-16. doi:10.1128/AAC.00115-16. PMC5278741 27872075

[40] Janardhanan J, Meisel JE, Ding D, Schroeder VA, Wolter WR, Mobashery S, Chang M 2016 *In vitro* and *in vivo* synergy of the oxadiazole class of antibacterials with β-lactams. Antimicrob Agents Chemother 60:5581–5588. doi:10.1128/AAC.00787-16. 27401567 PMC4997879

[41] Lepak AJ, Seiler P, Surivet JP, Ritz D, Kohl C, Andes DR 2016 *In vivo* pharmacodynamic target investigation of two bacterial topoisomerase inhibitors, ACT-387042 and ACT-292706, in the neutropenic murine thigh model against *Streptococcus pneumoniae* and *Staphylococcus aureus*. Antimicrob Agents Chemother 60:3626–3632. doi:10.1128/AAC.00363-16. 27044547 PMC4879394

[42] Vogelman B, Gudmundsson S, Turnidge J, Leggett J, Craig WA 1988 *In vivo* postantibiotic effect in a thigh infection in neutropenic mice. J Infect Dis 157:287–298. doi:10.1093/infdis/157.2.287. 3121761

[43] Zuluaga AF, Salazar BE, Rodriguez CA, Zapata AX, Agudelo M, Vesga O 2006 Neutropenia induced in outbred mice by a simplified low-dose cyclophosphamide regimen: characterization and applicability to diverse experimental models of infectious diseases. BMC Infect Dis 6:55. doi:10.1186/1471-2334-6-55. 16545113 PMC1434751

[44] Parsons JB, Frank MW, Jackson P, Subramanian C, Rock CO 2014 Incorporation of extracellular fatty acids by a fatty acid kinase-dependent pathway in *Staphylococcus aureus*. Mol Microbiol 92:234–245. doi:10.1111/mmi.12556. 24673884 PMC4007170

[45] Han X, Gross RW 1995 Structural determination of picomole amounts of phospholipids via electrospray ionization tandem mass spectrometry. J Am Soc Mass Spectrom 6:1202–1210. doi:10.1016/1044-0305(95)00568-4. 24214071

[46] Hsu FF, Turk J 2001 Studies on phosphatidylglycerol with triple quadrupole tandem mass spectrometry with electrospray ionization: fragmentation processes and structural characterization. J Am Soc Mass Spectrom 12:1036–1043. doi:10.1016/S1044-0305(01)00285-9.

[47] Mazzella N, Molinet J, Syakti AD, Dodi A, Doumenq P, Artaud J, Bertrand JC 2004 Bacterial phospholipid molecular species analysis by ion-pair reversed-phase HPLC/ESI/MS. J Lipid Res 45:1355–1363. doi:10.1194/jlr.D300040-JLR200. 15102893

[48] Bae T, Schneewind O 2006 Allelic replacement in *Staphylococcus aureus* with inducible counter-selection. Plasmid 55:58–63. doi:10.1016/j.plasmid.2005.05.005. 16051359

[49] Bose JL, Fey PD, Bayles KW 2013 Genetic tools to enhance the study of gene function and regulation in *Staphylococcus aureus*. Appl Environ Microbiol 79:2218–2224. doi:10.1128/AEM.00136-13. 23354696 PMC3623228

[50] Bligh EG, Dyer WJ 1959 A rapid method of total lipid extraction and purification. Can J Biochem Physiol 37:911–917. doi:10.1139/o59-099. 13671378

