# Supplementary material for: Host Fatty Acid Utilization by Staphylococcus aureus at the Infection Site
Source: mBio. 2020 May 19;11(3):e00920-20. doi: 10.1128/mBio.00920-20 (PMC7240157; doi:10.1128/mBio.00920-20)
Supplement: FIG S1 [file mBio.00920-20-sf001.pdf]

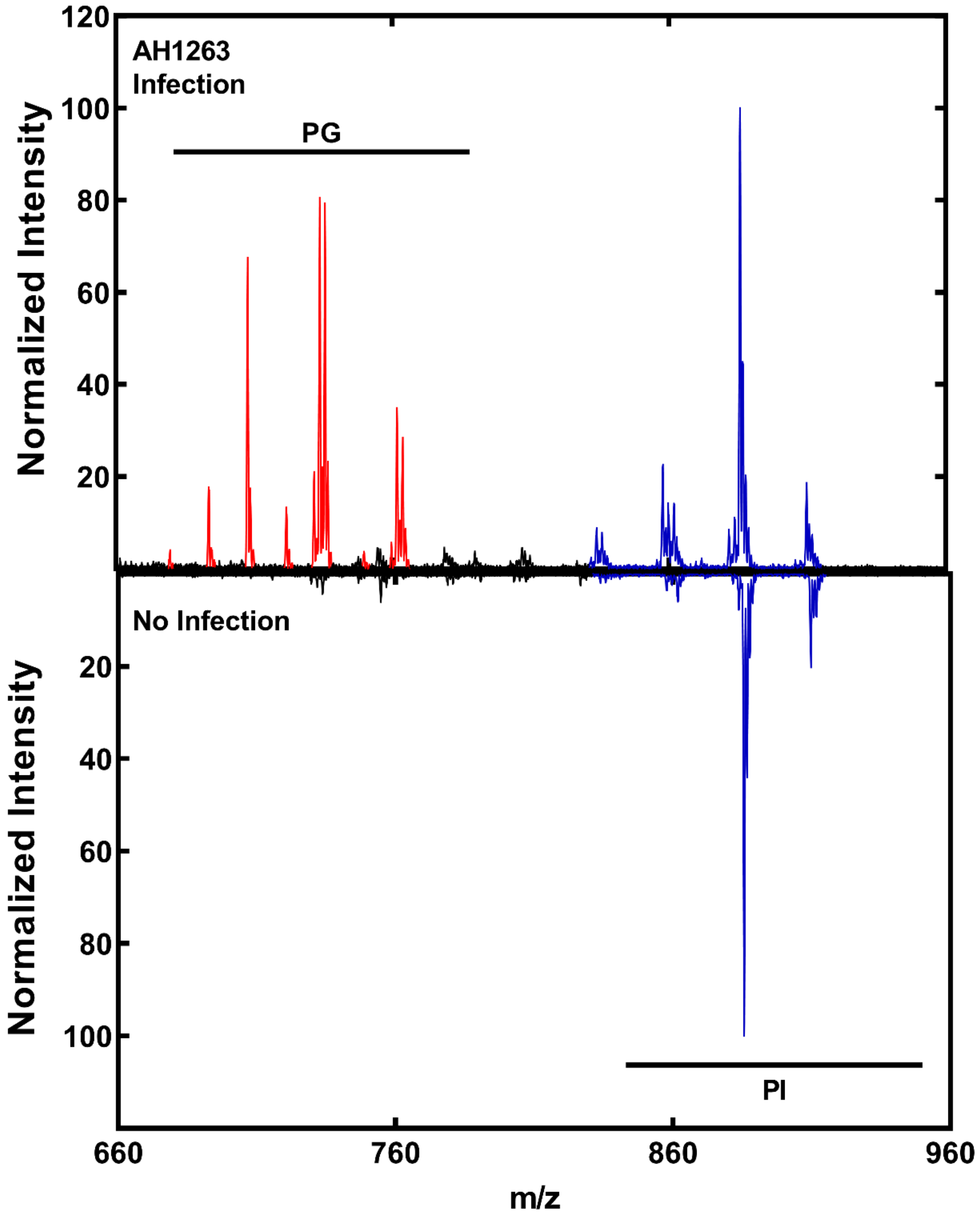

FIG S1 Reflection plot highlighting the *S. aureus* PG molecular species containing 15:0 FA in the infected thigh. The thigh lipid extract was enriched for PG and phosphatidylinositol (PI) using solid phase extraction, and the PG molecular species containing 15:0 were detected using mass spectrometry by scanning for parent ions that lose *m/z* = 241 corresponding to the loss of a 15:0 FA. Mouse PI was also detected because it copurified with PG and its fragmentation also gives a loss of *m/z* = 241 due to the removal of the inositol headgroup. The Upper Panel shows the *m/z* = 241 scan from a thigh infected with strain AH1263 (wild type). The Lower Reflection Panel shows the *m/z* = 241 scan from an uninfected thigh. The new PG molecular species peaks that appear in the *S. aureus* infected thigh are highlighted in red and the mammalian PI molecular species are shown in blue.
