# Supplementary material for: Host Fatty Acid Utilization by Staphylococcus aureus at the Infection Site
Source: mBio. 2020 May 19;11(3):e00920-20. doi: 10.1128/mBio.00920-20 (PMC7240157; doi:10.1128/mBio.00920-20)
Supplement: TABLE S1 [file mBio.00920-20-st001.pdf]

Table S1 Quantification of the PG molecular species distribution found in the PG of strains grown in the thigh model. Multiple spectra were obtained using the methods outlined in Fig. 3, the percentage of each peak was calculated and the percent distribution determined.

	% Total PG (Mean ± SD)					
PG Species	AH1263 (WT) Luria broth	AH1263 (WT) Thigh	JLB2 (Δ*fakA*) Thigh	JLB31 (Δ*fakB1* Δ*fakB2*) Thigh	JLB27 (Δ*fakB1*) Thigh	JLB30 (Δ*fakB2*) Thigh
30:0-PG	21.71 ± 2.40	3.16 ± 0.61	15.24 ± 1.58	13.60 ± 1.31	3.11 ± 0.51	2.97 ± 0.18
31:0-PG	0.64 ± 0.11	19.29 ± 1.44	13.55 ± 0.34	11.90 ± 0.95	7.22 ± 0.80	25.66 ± 1.92
32:0-PG	55.29 ± 2.52	4.91 ± 0.64	19.77 ± 2.00	21.11 ± 2.06	5.91 ± 1.48	5.54 ± 0.32
33:1-PG	ND[a]	21.58 ± 1.26	ND	ND	26.78 ± 0.92	ND
33:0-PG	7.70 ± 0.66	23.46 ± 0.85	24.32 ± 0.97	24.52 ± 1.32	15.19 ± 1.10	37.02 ± 1.41
34:0-PG	11.58 ± 0.90	3.86 ± 1.32	9.95 ± 2.75	13.25 ± 2.43	8.36 ± 0.93	3.60 ± 0.36
35:1-PG	ND	9.33 ± 0.91	ND	ND	21.76 ± 1.06	ND
35:0-PG	3.08 ± 0.43	14.41 ± 0.98	17.16 ± 2.49	15.63 ± .91	11.67 ± 0.95	26.64 ± 1.27

[a]ND means not detected
