# Supplementary material for: Host Fatty Acid Utilization by Staphylococcus aureus at the Infection Site
Source: mBio. 2020 May 19;11(3):e00920-20. doi: 10.1128/mBio.00920-20 (PMC7240157; doi:10.1128/mBio.00920-20)
Supplement: FIG S2 [file mBio.00920-20-sf002.pdf]

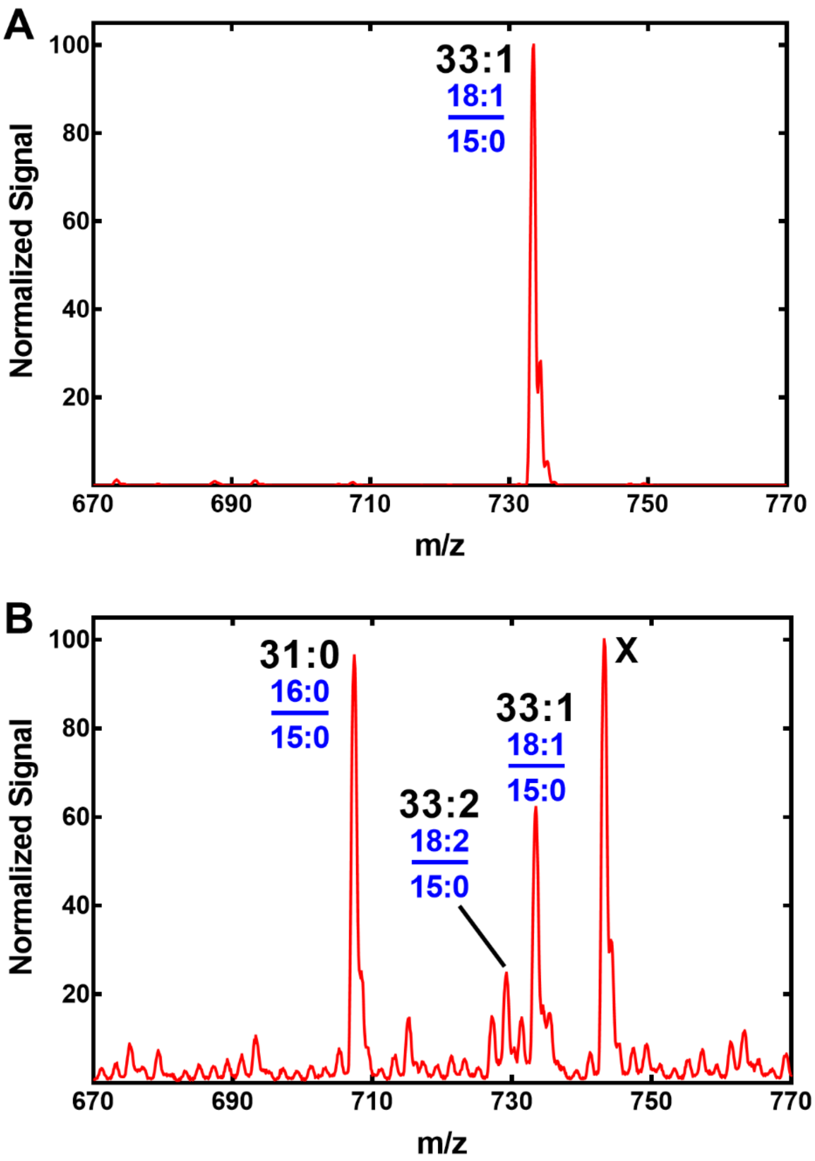

FIG S2 Host FA utilization by strain PDJ70 (Δ*plsX*). The identities of the FA in the 1- and 2-positions (blue) are shown for each molecular species (black). (A) A representative mass spectrum of the PG molecular species of strain PDJ70 (Δ*plsX*) used to inoculate the thigh grown in tryptone broth supplemented with 500 μM 18:1. (B) A representative mass spectrum of PG molecular species from strain PDJ70 (Δ*plsX*) recovered from the thigh infection site. The PG fraction was isolated, and the mass spectrum of the sample was generated using a *m/z* = 241 scan to detect PG molecular species containing 15:0 (See Fig. S1). The peak labeled "X" is not a PG molecular species.
