# Supplementary material for: Host Fatty Acid Utilization by Staphylococcus aureus at the Infection Site
Source: mBio. 2020 May 19;11(3):e00920-20. doi: 10.1128/mBio.00920-20 (PMC7240157; doi:10.1128/mBio.00920-20)
Supplement: TABLE S2 [file mBio.00920-20-st002.pdf]

Table S2 Bacterial strains and primers.

Strains	Relevant Genotype	Source
AH1263	USA300-0114	(51)
JLB2	Δ*fakA*	(8)
JLB27	Δ*fakB1*::ΦNΣ	(8)
JLB30	Δ*fakB2*	(8)
JLB31	Δ*fakB1*::ΦNΣ Δ*fakB2*	(8)
PDJ69	Δ*accD*	This study
PDJ70	Δ*plsX*	This study
